# The three-dimensional coupling mechanism in scoliosis and its consequences for correction

**DOI:** 10.1007/s43390-023-00732-8

**Published:** 2023-08-09

**Authors:** Lorenzo Costa, Tom P. C. Schlosser, Peter Seevinck, Moyo C. Kruyt, René M. Castelein

**Affiliations:** 1 Department of Orthopaedic Surgery, University Medical Centre Utrecht, Postbus 85500, G 05.228, 3508 GA Utrecht, The Netherlands; 2 Department of Imaging, University Medical Centre Utrecht, Utrecht, The Netherlands

**Keywords:** Adolescent idiopathic scoliosis, Scoliosis, Coupling mechanism, Extension

## Abstract

**Introduction:**

In idiopathic scoliosis, the anterior spinal column has rotated away from the midline and has become longer through unloading and expansion of the intervertebral discs. Theoretically, extension of the spine in the sagittal plane should provide room for this longer anterior spinal column, allowing it to swing back towards the midline in the coronal and axial plane, thus reducing both the Cobb angle and the apical vertebral rotation.

**Methods:**

In this prospective experimental study, ten patients with primary thoracic adolescent idiopathic scoliosis (AIS) underwent MRI (BoneMRI and cVISTA sequences) in supine as well as in an extended position by placing a broad bolster, supporting both hemi-thoraces, under the scoliotic apex. Differences in T4–T12 kyphosis angle, coronal Cobb angle, vertebral rotation, as well as shape of the intervertebral disc and shape and position of the nucleus pulposus, were analysed and compared between the two positions.

**Results:**

Extension reduced T4–T12 thoracic kyphosis by 10° (*p* < 0.001), the coronal Cobb angle decreased by 9° (*p* < 0.001) and vertebral rotation by 4° (*p* = 0.036). The coronal wedge shape of the disc significantly normalized and the wedged and lateralized nucleus pulposus partially reduced to a more symmetrical position.

**Conclusion:**

Simple extension of the scoliotic spine leads to a reduction of the deformity in the coronal and axial plane. The shape of the disc normalizes and the eccentric nucleus pulposus partially moves back to the midline.

## Introduction

The complex three-dimensional (3D) nature of adolescent idiopathic scoliosis (AIS) has consequences for reduction strategies, both surgically as well as conservatively [1, 2]. The anterior part of the spinal column in scoliosis of multiple aetiologies is longer than the posterior part, mainly due to anterior expansion of the intervertebral discs (IVD) [3–5]. In surgery, this is encountered as difficulty to obtain simultaneous vertebral derotation and thoracic kyphosis [6, 7].

Conservative treatment has stood the test of time, but focuses to a large extent on preservation of the coronal Cobb angle below a certain threshold value, usually the surgical limit of 40°–45° [8, 9].

Also, in conservative treatment, a thorough understanding of the coupling mechanisms between the different planes is important in order to obtain the best result. On theoretical grounds, extension of the scoliotic spine in the sagittal plane should provide room for the longer anterior column to swing back towards the midline, thus reducing the coronal plane Cobb angle as well as the axial plane vertebral rotation. In scoliosis, much of the deformity occurs in the IVD, which becomes wedge shaped with a lateralized nucleus pulposus [10–12]. It can be assumed that through the same extension manoeuvre, instantaneous reduction will take place in the disc with normalization of its initially wedged shape and a more symmetrical position of the nucleus pulposus, dependent on the structurality of the deformation.

Purpose of this study is to analyse the effect of simple extension of the spine in the sagittal plane on the magnitude of the coronal plane curvature as well as on the axial plane vertebral rotation, using novel BoneMRI technique. Furthermore, the changes in the shape of the disc as well as the shape and position of the nucleus pulposus inside the disc are studied.

## Materials and methods

### Study population

This prospective study was executed after IRB approval. Patients indicated to undergo an MRI scan as part of their workup for idiopathic scoliosis were recruited between November 2021 and December 2022. A total of ten female patients with AIS aged between 12–16, with primary thoracic curves were included in this study. Patients with non-idiopathic scoliosis (or with any additional spine pathology), lack of informed consent and general contra-indications for MRI scans were excluded from participating in this study [13]. Patients were approached at our outpatient scoliosis clinic and were asked to participate in this study. At that time, subject information was provided to the participants and parents/legal representatives and informed consent was signed. For demographics, Cobb angle and thoracic kyphosis in normal standing position were measured on the most recent radiographs, obtained less than 6 months before the MRI examination.

### Study procedure and MRI analysis

**Fig. 1:**
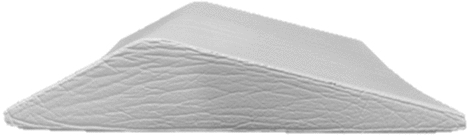
picture of the bolster used in the MRI scan to provide extension of the spine. The bolster is 9 cm height, 40 cm large and 45 cm in depth

Next to the standard MRI scan for detection of possible neural axis abnormalities, two additional MRI sequences with particular focus on the peri-apical area (defined as the apical vertebra, two vertebrae above and two below) of the thoracic curve, were added to the normal scan protocol [14]. We used coronal view “Volume Isotropic Turbo spine echo Acquisition”, in short cVISTA, to detect the 3D configuration of the IVD, and BoneMRI, a modern technique that uses different input images to detect bony structures via AI algorithms. Patients were positioned first in normal supine position (NSP) and then in extended supine position (ESP) by placing a broad MRI compatible bolster under the apex of the curve (Fig. 1). The cushion allowed the patient to lay comfortably in some extension, while both hemi-thoraces were equally supported with equal weight on both shoulders, as in the neutral position.

### Image analysis

*X-ray* Cobb angles of the main thoracic curves were measured using the standard Cobb method on the standing coronal plane image where the spine was best visible. Sagittal kyphosis angle was measured using the upper endplate of T4 and the lower endplate of T12 on the standing sagittal plane image. X-rays only served for demographic purposes as shown in Table 1.

**Table 1:** Demographic data are shown for the AIS cohort

Baseline statistic			
	*n* (%)	Mean (LCI; UCI)	SD
Age in years	10	14 (13; 15)	1.4
Risser's sign			
0	1 (10%)		
1			
2			
3	3 (30%)		
4	3 (30%)		
5	3 (30%)		
Coronal Cobb (standing X-ray)	10	53° (48°; 60°)	7.4
T4-T12 TK (standing X-ray)	10	28° (25°; 30°)	3.6
Lenke type (in one patient no bending X-rays were available)			
Type I	4 (44%)		
Type III	5 (56%)		

*SD* standard deviation, *TK* thoracic kyphosis, *LIC* lower confidence of interval, *UIC* upper confidence of interval. One patient did not have bending X-rays and was excluded by the baseline statistic in the Lenke type section

*MRI* for curve morphology analyses, an in-house developed software package (ScoliosisAnalysis 4.1) and PACS 23.1.10.4570 (Sectra AB, Linkoping, Sweden) were used to reconstruct the coronal, sagittal and axial plane images in both positions. The coronal Cobb angle, the sagittal T4-T12 kyphosis angle and the axial vertebral rotation were measured in both positions. Furthermore, the 3D shape of the intervertebral disc as well as the shape and position of the nucleus pulposus was analysed.

**Fig. 2:**
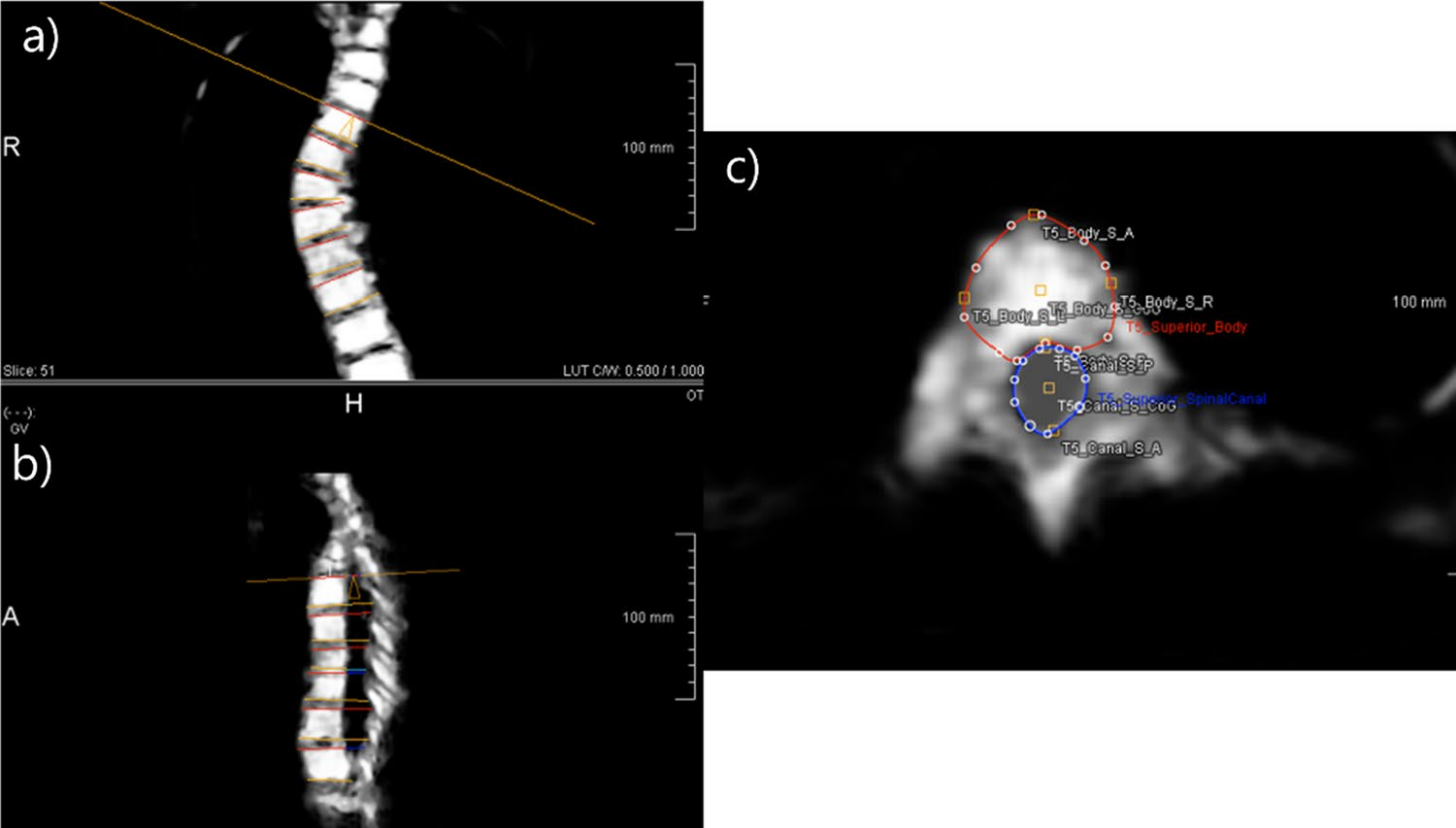
the orientation of the upper and lower endplate of each vertebra within the curve was determined correcting for the coronal (**a**), sagittal plane (**b**) and axial plane (**c**). The true axial plane was thus determined. The observer drew the contour of the vertebral body (red out line) and spinal canal (blue outline) as seen in picture c. The software automatically determined the most anterior and posterior points and the COVs of both vertebral body and spinal canal, thus providing an axis per vertebra

The axial rotation of the apex, two vertebrae above, two below and first distal neutrally rotated vertebra not involved in the scoliotic curve, identified at MRIs, were measured. For this, the 3D orientation of superior and inferior endplates of the peri-apical area and the first neutrally rotated vertebra were defined on multiplanar reconstructions. On the true transverse sections of each endplate, adjusted in all three planes, the endplate and spinal canal were segmented. This enabled to automatically detect each anterior–posterior axis (Fig. 2) [15]. Axial rotation per endplate was defined as the angle between each vertebral endplate’s AP axis and the AP axis of the lower endplate of the first distal neutrally rotated vertebra.

For IVD measurements, Mimics and 3-Matics (Materialize NV, Leuven, Belgium) were used for segmentation and automatic measurements of the IVDs and NPs.

**Fig. 3:**
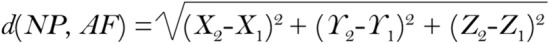
formula to detect the distance (*d*) between two points knowing their coordinates (*x*, *y*, *z*)

The discs of the apical region (defined as the apical vertebra or disc and the two adjacent vertebrae and discs) were segmented in their two components, namely nucleus pulposus (NP) and annulus fibrosus (AF)) via Mimics. NP plus AF represented the total IVD. Thus, the segmented discs were imported in 3-Matics and the coordinates of the centres of volume (CoV) were found. Finally, via the formula shown in Fig. 3, the distance between the CoV of NP and the CoV of AF of the same IVD was determined.

**Fig. 4:**
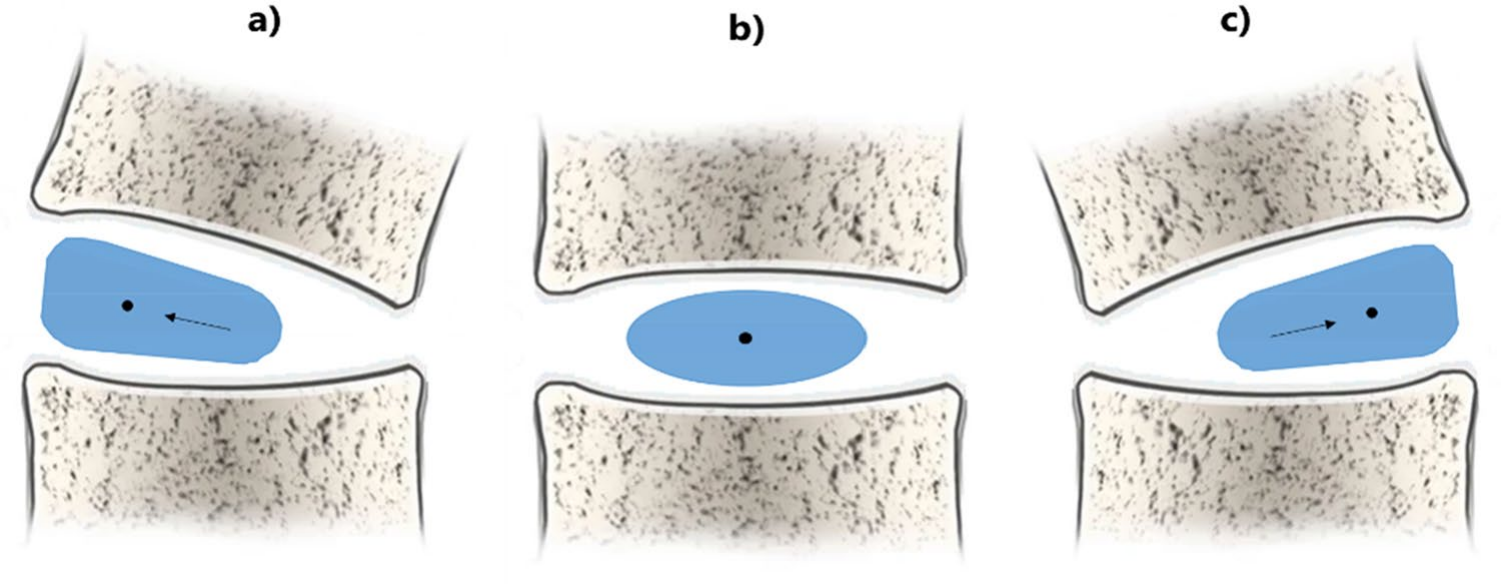
Representation of the shape of the IVD (in blue) in the coronal view and the position of the COV (black dot) in left (**a**) or right (**c**) convexity during scoliosis and in normal spine (**b**). The arrows show the shift to the left (**b**) or to the right (**c**) of the COVs in different NPs shapes. It is assumed that a central COV represents a uniform distribution of the volume within the object and therefore a more symmetrical shape in the coronal view

As the CoV represents the centre of volume, analysing the distance in the mid-coronal plane (left end- CoV and right end- CoV distances), we can see the distribution of the volume in the coronal view. If the CoV is positioned in the middle of the object (image 4b), the distance between the CoV and the two endpoints is equal. In this case, the object has a symmetrical shape. Vice versa, if the CoV is not positioned in the middle, the distances differ and the object is not symmetrical, such as in an IVD deformed by scoliosis (Fig. 4). Therefore, to understand the distance from the CoV to the borders in the mid-coronal plane, 3-matics was used. First, the CoVs of IVDs and NPs were found as previously discussed and the mid-coronal plane was created. Second, the most right and left points of the IVDs and NPs in the mid-coronal plane were determined automatically. Third, in the coronal plane, the distance from the concave and the convex lateral borders to the CoV was determined. The difference between the two distances (concave- CoV distance (EC) minus convex- CoV distance (XC)) was calculated.

These analyses were performed in the two different positions (NSP and ESP).

### Statistical analysis

The statistical analysis was performed using SPSS 22.0 (SPSS, Inc., Chicago, IL, United States). Descriptive baseline statistics for the demographics was calculated, providing mean, standard deviation (SD), lower confidence interval (LCI), and upper confidence interval (UCI). Before testing, normality of distribution of the curve severity (Cobb angle) in the population was verified using Q-Q plots. Paired t test was used to calculate the differences between the two variables (NSP and ESP). Pearson’s correlation test was used to calculate the linear relationship between the independent variables (NSP) and the dependent variables (ESP). As in the first aim, 4 different outcomes were analysed (TK, Cobb, vertebral rotation, and apical vertebral rotation), a p value less than 0,05, after Bonferroni correction, was considered statistically significant.

## Results

### Study population

Ten patients were included in this study. Patient characteristics are shown in Table 1. *Q*–*Q* plot revealed that the sample was normally distributed.

### 3-D morphology of the spine in NSP compared to ESP on BoneMRI scans

From the X-ray in standing position to NSP, TK reduced from 28° to 23° (− 18%) and coronal Cobb angle from 53° to 45° (− 15%). The mean peri-apical rotation was 11°, and rotation at the apex was 16°. In ESP, TK was reduced to 13°, the coronal Cobb angle was reduced to 36°, axial rotation to 7° and apical rotation to 9°.

**Fig. 5:**
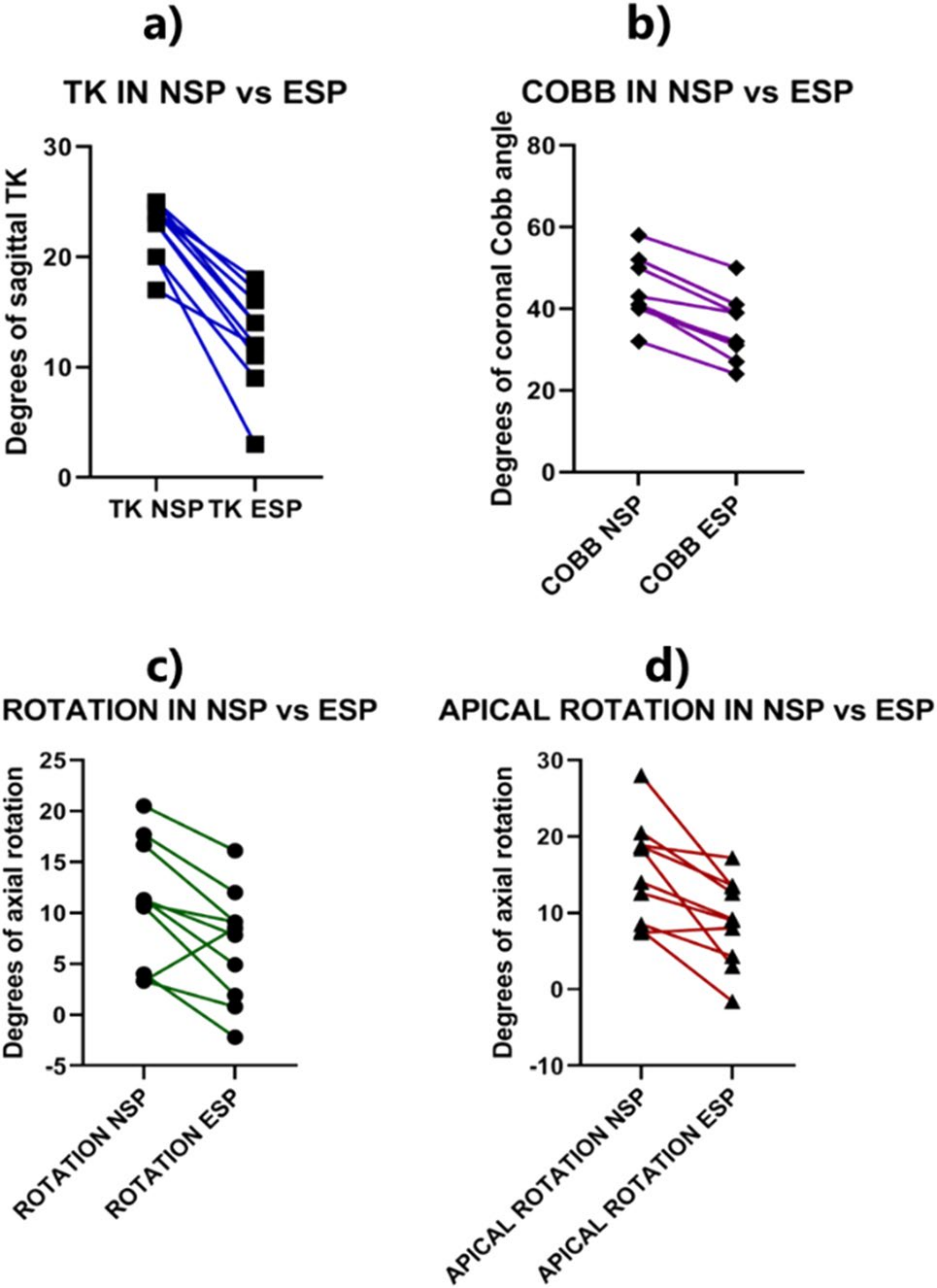
Line graph showing **a** reduction of kyphosis; **b** reduction of coronal Cobb angle; **c** mean de-rotation of all vertebrae; **d** mean de-rotation of the apical vertebrae

In Table 2 and Fig. 5, differences between the two groups are shown.

**Table 2:** Differences between the mean in NSP and in ESP

	NSP (SD)	ESP (SD)	Δ% NSP vs ESP	*P* value
Kyphosis	23° (2.6)	13° (4.4)	− 44%	< 0.001
Coronal Cobb	45° (7.8)	36° (7.8)	− 20%	< 0.001
Peri-apical rotation	11° (6.1)	7° (5.5)	− 37%	0.036
Apical rotation	16° (6.6)	9° (5.7)	− 42%	0.012

*SD* standard deviation, *Δ%* difference in percentage. *P* value was considered significant when < 0.01

### 3-D morphology of the IVD and NP in NSP and ESP on c-VISTA MRI scans

**Fig. 6:**
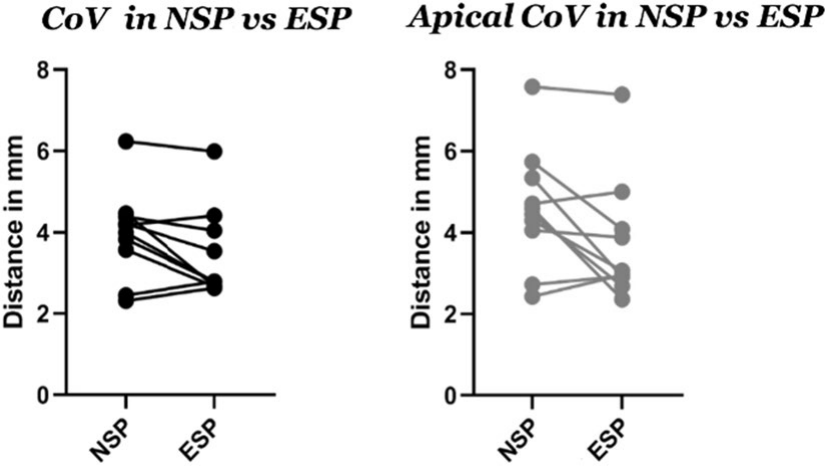
mean reduction of the distance between the COVs of both NP and AF. Left image represents the overall distance. Right image focuses on the apical region

The distance between the CoVs of the NP and the IVD, as a measure of lateralisation of the NP within the disc, was analysed throughout the apical and peri-apical areas of the curve. The mean distance in NSP was 4 mm (SD 1.6). In ESP, the mean was 3.5 mm (SD 1.5), which resulted to be different but not statistically significant from the mean distance in NSP (*P* value 0.108). At the apex the difference showed means of 4.6 mm (SD 1.6) and 3.7 mm (SD 1.5) but not statistical significance, respectively (*P* value 0.056) (Fig. 6).

**Fig. 7:**
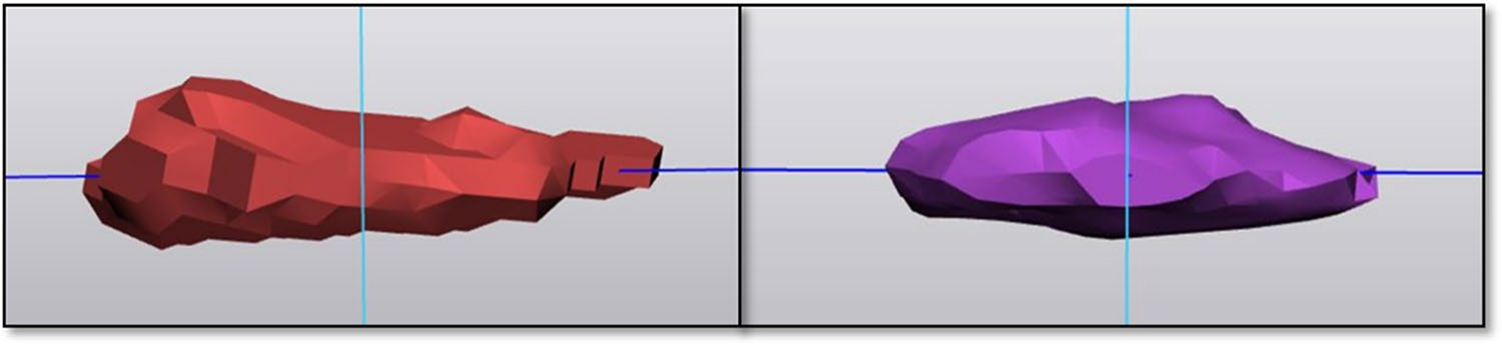
NP in NSP (left image) and ESP (right image)

Moreover, the distance from the CoVs to the lateral borders of the IVDs and NPs in the mid-coronal plane was analysed, as a measure of centralization in the coronal view (Fig. 7). Table 3 presents the difference between XC and EC, showing that in ESP, the NP and IVD have a more equal distance from the CoV to the lateral ends in the mid-coronal plane.

**Table 3:** The mean of the difference in distance in mm between XC and EC in both IVD and NP

	Mean	SD	LIC	UIC	*P* value
IVD					
NSP	2	1.2	3.9	0.3	0.064
ESP	1.3	0.8	3.1	0.1	
NP					
NSP	2.1	1.1	3.8	0.1	0.012
ESP	1.1	0.9	3.3	0.1	

*SD* standard deviation, *TK* thoracic kyphosis, *LIC* lower confidence of interval, *UIC* upper confidence of interval, *XC* convex-centroid distance, *EC* concave-centroid distance

### Correlation between the 3D changes of the curve

Pearson’s correlation analysis revealed no significant correlation between TK reduction and axial derotation (0.447, *p* value 0.78) and a very strong positive correlation between Cobb angle reduction and axial derotation in both overall peri-apical area and apex (respectively, 0.804 (*p* value 0.02) and 0.872 (*p* value 0.004)). TK and Cobb reduction revealed a strong positive correlation (0.796 (*p* value 0.024).

## Discussion

In this study, the 3D response of the AIS spine to apical extension was studied.

Furthermore, it aimed to shed light on the changes of the IVD during the extension of the spine and lastly, correlated the reduction of the kyphosis with the changes in the two other planes. For this experiment 3D T2 MRI and Bone MRI were used. The latter is a newly developed MRI sequence that enables the creation of a synthetic CT, based on AI algorithms, which permits an optimal evaluation of both the bony structures, as well as soft tissues such as IVDs, one of the most important stabilizers in the spine [16–18]. This is the first study that uses this MRI technique to analyse a scoliotic spine.

Extension of the spine led to a significant reduction of both the Cobb angle and the axial vertebral rotation, the latter both throughout the curve and around the apex. Furthermore, a strong correlation was found between the reduction of the coronal Cobb angle and the amount of axial derotation. This correlation was also found in other recent studies [19, 20].

**Fig. 8:**
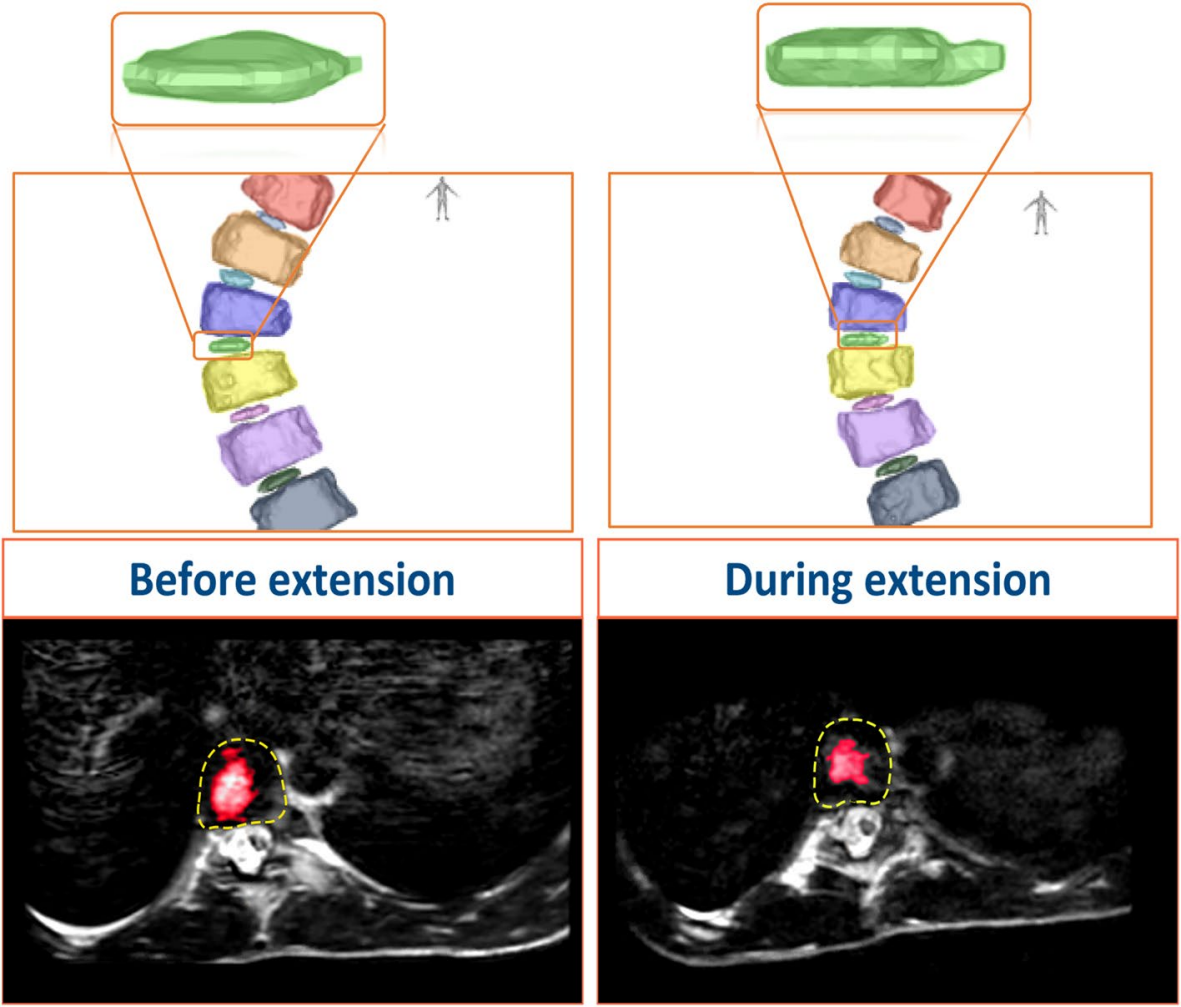
segmentation (above) and MRI in the axial plane (below) of the same patient in NS (left) and EP (right) positions

Furthermore, this study focused on the IVD as they are essential players in the stability of the spine, as well as the most deformed structures in scoliosis [4, 5, 11, 21, 22]. The distance between the CoV of the NP and the CoV of the overall IVD significantly differed between the two positions. The distance between the CoV of the NP and the CoV of the IVD decreased in ESP indicating that the NP re-positioned more evenly and partially returned to the centre of the IVD (Fig. 6). Moreover, centralization (Table 3) in the coronal plane was observed for both NP and IVD, suggesting a more uniform distribution of the volume in the coronal plane resulting in a more coronal symmetrical shape of both structures (Figs. 7 and 8).

The changes in the sagittal (intervention), coronal, and axial planes showed a clear relationship, which confirms the theory of the coupling between the three planes [23, 24]. Van Loon et al. presented similar coronal data on X-rays, when an extension of the thoracolumbar section of the spine resulted in a decrease between 31 and 36% of the coronal Cobb angle in adolescent scoliotic patients [23, 24]. Our study showed similar results giving a reduction of the coronal Cobb angle of 32%. In addition, as scoliosis is a 3D deformity, our analysis focused on the other two planes, showing reduction of the axial rotation of the vertebrae and centralization of the NP, as consequence of a direct reduction of the TK.

Our study demonstrates that an intervention in one plane in scoliosis leads to a response in the other planes. One could argue that the overall amount of 3-D deformation does not really change, but it can vary between the planes. An increase in the sagittal plane deformity (hypokyphosis) leads to a reduction of the deformity in the other two planes, indicating that the overall 3D deformity has not changed much but has moved from one plane to the other. Although thoracic hypokyphosis is certainly an undesired outcome in surgical scoliosis treatment, with proven complications in the junctional zone between the fixed and rigid and the more mobile spinal segments, the question is how important a decrease in thoracic kyphosis is in a mobile spine in a young individual [25]. In a partly fixed spine after scoliosis surgery, it has been shown to be related to junctional decompensation but in a mobile spine different compensation mechanisms occur, an important one being a gradual increase in positive sagittal balance over time [26, 27]. It could be argued that in conservative scoliosis treatment, it is more important to reduce the coronal and axial plane deformity, thereby restoring the symmetry in the disc, at the expense of a reduced thoracic kyphosis, leading to a more harmonious loading of the spine and an interruption of the deformity increasing vicious cycle in both the bone as well as the discs. Nevertheless, extension of the thoracic spine might cause potential negative effect such as neck pain, a reduction of the AP diameter of the chest, decreasing the space available for the airways and mediastinum, although our data show a derotation of the spine out of the convex hemi-thorax, possibly increasing the space available for the lungs [28, 29]. It seems logical, but this is a hypothesis that was not tested in this study, that the thoracic kyphosis will increase spontaneously over time, given the natural history of sagittal balance [30]. Unfortunately, valuable data regarding the persistence or significant changes of sagittal plane alignment after bracing at the end and beyond adolescence are currently lacking. This knowledge is crucial for effectively improve management and treatment of young patients with scoliosis, necessitating further investigation in this area.

This is the first study that focuses on the 3D changes in both bone and disc, that occur during extension of the scoliotic spine. Its strength lies in the very accurate 3D measurements that could be performed on both the bony vertebral bodies as well as the intervertebral discs including the shape and position of the NP via new analytical techniques. Bone MRI images allowed accurate assessment of the bony orientation, while c-Vista allowed analysis of the shape of the disc and the position of the nucleus pulposus within it.

Nevertheless, this study also has limitations. First, it is a proof of principle study, and the sample size is small, although the changes in the coronal and transverse planes are fairly evident, consistent and significant. Second, it involves patients with relatively large curves, because these were the ones indicated for MRI as part of their general work up. Since an excess of anterior length is a uniform characteristic of different types of scoliosis, we do believe, however, that coupling principles are irrespective of relatively arbitrary cut-off values of Cobb angles. Third, imaging in this study focused only on the scoliotic section of the main thoracic curve, not considering the whole spine. Reason for this was that MRI scanning for the aims of this study is time consuming because of the necessity to obtain both bony and soft tissue details. Visualization of the whole spine would have increased the scanning time significantly, increasing the burden on our patients. Finally, this study set out to demonstrate a general coupling principle of the scoliotic spine. It does not address the implications this may have for conservative treatment, that could be the topic of a future study.

## Conclusion

This study describes the 3D mechanism of action of the spine under extension in patients with primary thoracic AIS. Extension on the apical area of the scoliotic curve provides reduction of the thoracic kyphosis with simultaneous reduction of the coronal Cobb angle and derotation in the axial plane. The initially wedge-shaped intervertebral disc returns to a more symmetrical shape, whereas the nucleus pulposus reduces out of its lateralized position to a more centralized one. Although the ultimate effect of reduction of the thoracic kyphosis remains to be determined, we believe that natural evolution of sagittal balance during ageing suggests that this could be favourable. This knowledge could be incorporated in the conservative treatment of scoliosis, analogous to the treatment of clubfoot, where reduction of the deformity in one plane is facilitated by making room in another plane.

## Data Availability

As the data is privacy-sensitive, data will be available under restriction by sending an email to the corresponding author.
