# Correction: The three-dimensional coupling mechanism in scoliosis and its consequences for correction

**DOI:** 10.1007/s43390-023-00776-w

**Published:** 2023-10-13

**Authors:** Lorenzo Costa, Tom P. C. Schlosser, Peter Seevinck, Moyo C. Kruyt, René M. Castelein

**Affiliations:** 1 Department of Orthopaedic Surgery, University Medical Centre Utrecht, Postbus 85500, G 05.228, 3508 GA Utrecht, The Netherlands; 2 Department of Imaging, University Medical Centre Utrecht, Utrecht, The Netherlands

**Correction: Spine Deformity** 10.1007/s43390-023-00732-8

The authors of the article would like to state the following acknowledgment:

